# Are COVID-19 Polymorphisms in ACE and ACE2 Prognosis Predictors?

**DOI:** 10.3390/cimb46080480

**Published:** 2024-07-28

**Authors:** Fabiana Amaral Guarienti, Fernando Antônio Costa Xavier, Mateus Duarte Ferraz, Fernanda Wagner, Daniel Rodrigo Marinowic, Jaderson Costa da Costa, Denise Cantarelli Machado

**Affiliations:** 1 Post Graduation Program of Geriatrics and Gerontology, Pontifical Catholic University of Rio Grande do Sul, Porto Alegre 90619, Brazil; fabiana.guarienti@pucrs.edu.br (F.A.G.); daniel.marinowic@pucrs.br (D.R.M.); dcm@pucrs.br (D.C.M.); 2 Laboratory of Molecular Biology and Immunology, Brain Institute of Rio Grande do Sul, Porto Alegre 90610, Brazil; mateus.ferraz@edu.pucrs.br; 3 Post Graduation Program of Medicine and Health Sciences, Pontifical Catholic University of Rio Grande do Sul, Porto Alegre 90619, Brazil; fernanda.wagner002@edu.pucrs.br (F.W.); jcc@pucrs.br (J.C.d.C.); 4 Laboratory of Precision Medicine and Biotechnology, Brain Institute of Rio Grande do Sul, Porto Alegre 90610, Brazil; 5 School of Medicine, Pontifical Catholic University of Rio Grande do Sul, Porto Alegre 90619, Brazil; 6 Laboratory of Neurosciences and Electrophisiology, Brain Institute of Rio Grande do Sul, Porto Alegre 90610, Brazil

**Keywords:** polymorphism, COVID-19, ACE, prognosis, SARS-CoV-2

## Abstract

Regardless of the containment of the SARS-CoV-2 pandemic, it remains paramount to comprehensively understand its underlying mechanisms to mitigate potential future health and economic impacts, comparable to those experienced throughout the course of the pandemic. The angiotensin-converting enzyme 2 (ACE2) provides anchorage for SARS-CoV-2 binding, thus implicating that ACE and ACE2 might contribute to the variability in infection severity. This study aimed to elucidate predisposing factors influencing the disease course among people infected by SARS-CoV-2, focusing on angiotensin-converting enzyme (ACE) and ACE2 polymorphisms. Notably, despite similar demographics and comorbidities, COVID-19 patients exhibit substantial differences in prognosis. Genetic polymorphisms in ACE and ACE2 have been implicated in disease progression, prompting our investigation into their role in COVID-19 evolution. Using next-generation sequencing (NGS), we analyzed ACE and ACE2 genes in a sample group comprising six subjects infected by SARS-CoV-2. Our findings revealed a correlation between specific polymorphisms and COVID-19 outcomes. Specifically, ACE and ACE2 intronic deletions were observed in all deceased patients, suggesting a potential association with mortality. These results highlight the significance of genetic factors in shaping the clinical course of COVID-19, emphasizing the importance of further research into the impact of genetic variations on COVID-19 severity.

## 1. Introduction

The coronavirus pandemic in 2019 affected several countries around the world, generating more than 6 million deaths, with much of the consequences resulting from multisystemic dysfunctions such as severe acute respiratory syndrome, kidney failure, progressive changes in inflammatory factors and multiple organ failure [1]. The causative agent of Coronavirus respiratory syndrome 2 (SARS-CoV-2) is a virus transmitted mainly via droplets in the air, thus generating COVID-19 in humans, which was first identified at the end of the year 2019 in Wuhan in China [2]. Through a large task force action, including research groups from diverse countries around the world, several risk factors were identified representing a major impact that led to an increase in morbidity in COVID-19 patients, including advanced age, male gender, pre-existing comorbidities, and racial/ethnic disparities, with age being the most influential factor.

COVID-19 cases may vary in symptoms, characterized as asymptomatic, mild, moderate, severe, and critical. “Asymptomatic” indicates patients who were verified with positive laboratory tests for COVID-19 and yet showed an absence of symptoms. “Mild” is characterized by the presence of non-specific symptoms, such as cough, sore throat or runny nose, followed or not by anosmia, ageusia, diarrhea, abdominal pain, fever, chills, myalgia, fatigue and/or headache. “Moderate” refers to the most frequent symptoms that can range from mild signs of disease, such as persistent cough and daily persistent fever, to signs of progressive worsening in another symptom related to COVID-19 (adynamia, prostration, hyporexia and diarrhea) in addition to the presence of pneumonia and no serious signs or symptoms. With regard to “severe” symptoms, severe acute respiratory syndrome is considered (a flu syndrome that presents dyspnea/respiratory discomfort or persistent pressure in the chest, or oxygen saturation less than 95% in room air, or cyanosis of the lips or face). “Critical” involves the following main symptoms: sepsis, acute respiratory distress syndrome, severe respiratory failure, multiple organ dysfunction, severe pneumonia, the need for respiratory support and admissions to intensive care units. The individual’s genetic predisposition can also be considered an important issue to SARS-CoV-2 infection, with its variations and degrees of severity related to the renin–angiotensin–aldosterone system (RAAS). In this context, genetic abnormalities (polymorphisms, mutational changes in sequencing genes) have raised questions about their possible consequences in the prognosis of COVID-19. Polymorphisms can occur in coding and non-coding gene sequences, both in introns and exons, and can lead to qualitative and quantitative changes in proteins.

The presence of insertions or deletions in the angiotensin-converting enzyme (ACE) gene may affect ACE expression levels. The deletion/deletion (DD) genotype is associated with elevated levels of renal ACE when compared to subjects with the genotype insertion/insertion (II). Most studies confirm that the ACE I/D polymorphism is involved in the evident susceptibility of nephropathy, whereby type II plays a protective role against type 1 and type 2 diabetes. The most common ACE polymorphism occurs in intron 16 of the ACE gene and consists of two alleles: deletion (D) and insertion (I) [3].

According to Delanghe et al., 2020, after analyzing data from patients with COVID-19 in April 2020 in 33 countries in Europe, Africa and the Middle East, the ACE gene polymorphism caused changes in intratissue and serum ACE concentrations [4]. Gene deletion (DD) was associated with reduced ACE2 expression. Through ACE2, SARS-CoV-2 enables its connection with the cell, thus facilitating viral replication.

Montgomery and collaborators detected cardiovascular evidence related to left myocardial hypertrophy, which occurred only in individuals with the DD genotype. The authors concluded that exercise-induced left myocardial enlargement in men was strongly associated with the ACE I/D polymorphism [5]. Genetic polymorphisms like ACE rs4646994, ACE2 rs2285666 GG, TMPRSS2 rs12329760 CC and the presence of the C allele may serve as predictive models for the severity of COVID-19 [6,7,8]. 

Gallo et al. (2021) demonstrated that prevalence studies indicate the general frequency of the D allele at 54% when there is no relationship to gender, but found differences in ethnicities. In the African American population, the deletion polymorphism is associated with increased systolic blood pressure, hypertension, and altered vascular reactivity with potential impact on cardiovascular disease [9]. However, in Arab populations (Egyptians, Jordanians and Syrians), D allele frequencies are approximately 65%. Consequently, these ethnicities would present a tendency towards a higher incidence of cardiovascular diseases and resistance to therapy with ACE inhibitors.

Studies indicate that among patients infected with SARS-CoV-2 who died, those who were verified with having disseminated intravascular coagulation (DIC) presented the production of high levels of D-dimers and other products of fibrin degradation, corroborating the relationship between the genetic profile of individuals and the severity of the disease [10].

## 2. Materials and Methods

### 2.1. Participants and Sample

The Informed Consent Form was applied to all participants and the project was approved by the PUCRS Research Ethics Committee with No. 3.977404. For our sample, a total of 6 patients were analyzed, with ages ranging from 50.9 to 80.4 years. Only participants who tested positive for SARS-CoV-2 by real-time PCR test (RT-qPCR) on the first to second day of their arrival at the hospital and who were hospitalized at the ICU unit were included. Participants were hospitalized in the intensive care unit at São Lucas Hospital in Porto Alegre, Brazil, between March and June of 2020. In order to participate in the study, patients had to test positive for SARS-CoV and be hospitalized. These subjects were separated into subgroups as recommended by the World Health Organization. They were separated into groups of moderate, severe, and critical illness, according to criteria recommended by the Ministry of Health already set out in the introduction.

An informed consent form was applied to all patients, which was read by the attending physician and the procedures to be followed were explained, according to the research protocol. Blood was collected peripherally by a qualified team member trained for the procedure. After collection, the samples were placed in temperature-controlled boxes and immediately transported to the Cellular and Molecular Biology Laboratory at the Brain Institute (InsCer). In the laboratory, blood samples were processed to separate serum and leukocytes. Leukocytes were preserved in RNAlater (Sigma-Aldrich, San Louis, MO, USA). Both serum and leukocytes were stored in an ultrafreezer (−80 °C) until analysis. In total, our sample consists of 6 subjects, and the next-generation sequencing (NGS) technique was performed to detect ACE and ACE2 polymorphisms in a sample of 6 recently diagnosed patients. A mononuclear fraction of the blood of these patients was obtained, which was subsequently separated and stored at −80 °C in RNAlater.

### 2.2. DNA Extraction

DNA extraction was carried out using the ReliaPrep Blood gDNA Miniprep System (Promega, Madison, WI, USA). Samples were quantified by fluorometry (Qubit 2.0—Thermo Fisher Scientific, Waltham, MA, USA) and sample purity was determined by spectrophotometry (NanoDrop—Thermo Fisher Scientific), whereby only samples with a A260/A280 ratio between 1.8 and 2.0 were considered pure. The extracted DNA were then stored in a −2 °C freezer until used later.

### 2.3. Library Preparation

Libraries were prepared through PCR on the IonChef (Thermo Fisher Scientific) according to the manufacturer’s instructions. Libraries were quantified by qPCR accordingly and then diluted to reach a concentration of 100 pM. Once this was performed, the samples were prepared for clonal amplification and subsequent sequencing on the IonTorrent S5 system (Thermo Fisher Scientific).

### 2.4. Next-Generation Sequencing (NGS) and Data Analysis

Two genes of interest were selected (ACE and ACE2). For the analysis, a depth of 1000× was considered. A workflow was created in the IonReporter System from Thermo Fischer Scientific to perform variant-calling. Data analysis was performed using the Thermo Fisher’s Ion Reporter Software version 5.2.0, comparing with the human genome GRCh37. Mean depth was of 2726.5×.

## 3. Results

### 3.1. Angiotensin-Converting Enzyme (ACE) Polymorphisms

Demographic data are shown in Table 1. We can see that among our enrolled patients, the moderate disease group (*n* = 1) presented 13 mutations for the ACE gene (Table 2). The patient in the moderate group demonstrated four exonic ACE mutations, all of which were classified as SNVs. Patients in the severe group (*n* = 3) presented 18 mutations in the ACE gene in total. Of the 18, 4 were exonic single-nucleotide variants (SNVs) and the remaining mutations were intronic (14), of which 3 were indels.

Among the severely ill patients who died, one had an intronic INDEL mutation (S03), while the other had two intronic deletions in ACE (S02) (Table 2). The group of critical patients, represented by S08 and S05 (*n* = 2), presented one polymorphism in ACE, which presented only in the intronic deletion group. In relation to the patient who died in the critical group (S05), the ACE gene had a deletion in the intron. Three patients (two severe and one critical) died. All had a deletion in an ACE intron (chr17:61571516—NM_000789.4(ACE):c.2306-19G>C).

### 3.2. Angiotensin-Converting Enzyme 2 (ACE2) Polymorphisms

All ACE2 polymorphism events found in our sample (a total of six patients) were presented in two patients, who died (Table 3). The moderate group did not present ACE2 polymorphisms, while the positive group for ACE2 polymorphisms included only one severe patient and the other critical. The patient in the severe group presented four polymorphisms in ACE2, of which three SNVs and one deletion (intronic). Of the SNVs found, there were one exonic and two intronic. 

Surprisingly, the other patient who died presented this same intronic deletion, which is the only common change in ACE2 between two patients who died. This issue represents that possibly this deletion, even in an untranslated region of the gene, could influence impact changes in gene expression. The ACE2 event found in common in both patients was an intron deletion polymorphism, at location chrX15589925. The other events found were SNVs in intron and exon.

## 4. Discussion

Several studies shave demonstrated strong associations between ACE-insertion/deletion (I/D) and COVID-19 [11,12]. ACE-D/D carriers have higher blood levels of ACE, approximately twice when compared to ACE-I/I individuals, and have been associated with hypertension, ARDS, and in-hospital mortality [13]. Therefore, the deletion allele is associated with COVID-19 progression [14] and SARS-CoV-2 infection rate and mortality, while the ACE-I/I genotype negatively correlates with infection rate and mortality [15]. Furthermore, data show that COVID-19 susceptibility may be associated with ACE I/D polymorphisms [16]. However, a meta-analysis (48,758 healthy subjects from 30 different countries) significantly associated ACE-I/D allele frequency ratio with the increase in the recovery rate, but not with mortality [17]. The key role played by ACE2 and ACE, in the regulation of the RAAS, has led researchers to launch the hypothesis that genetic polymorphisms may alter the activity and/or expression of these enzymes, suggesting that people who share these genetic alterations may have increased susceptibility to COVID-19 and SARS-CoV-2 infection [18].

Polymorphisms in ACE may contribute to this reduction in the immune response, through a deregulation of ACE activity, which would cause a deregulation of angiotensin II and a decrease in the activity of angiotensin1-7 [19]. The presence of several intronic polymorphisms in ACE was observed in patients who died. We highlight the AGT/A deletion intronic ACE polymorphism found at position chr17:61571516, given that this is the only ACE polymorphism presented by all patients who died. Such polymorphisms can cause an increase in ACE activity through the mutation of iRNAS (interfering RNAs) which provides mechanisms for post-transcriptional gene regulation.

ACE2 polymorphisms may also play a significant role in COVID-19 severity [20,21] and increase susceptibility to Long Covid syndrome [22]. Through genotyping of 550 patients, Möhlendick et al. (2021) demonstrated that the ACE2 polymorphism rs2285666 and also ACE2 rs2074192 [23] were implicated in a twofold increase in risk of SARS-CoV-2 infection and a threefold increase in COVID-19 severity and mortality. In contrast, the authors found no association between severity and infection risk with ACE polymorphism [20]. In another study, the same polymorphism mentioned above was also found, and another significantly increased the risk of developing a more severe SARS-CoV-2 infection, including the need for hospitalization [21]. 

According to the data found in the present study, four events of ACE2 polymorphisms occurred only in patients who later died. Two deceased patients shared the same INDEL polymorphism CAAAAAAAG/CAAAAAA at the intronic position chrX:15589925 of the ACE2 gene. Most of the mutations found are intron-related, which bring to notice the importance of these regions, formerly known as junk DNA. As far as the authors know, none of the polymorphisms reported in the present manuscript have a rsID, hence why it was not used to identify the polymorphisms found.

While the limited sample size of this study may raise concerns over its power, it is important to notice that the data presented here are in accordance with other studies present in the literature. The current report demonstrates that ACE and ACE2 intronic polymorphisms may play a decisive role in disease prognosis and could be further considered as possible predictors of COVID-19 severity and prognosis. Information about patients’ ACE and ACE2 polymorphism statuses could better guide the healthcare and management of SARS-CoV-2-hospitalized patients.

## Figures and Tables

**Table 1:** Demographic data.

Characteristics	All Participants (*n* = 6)	Total ACE Polymorphisms	Total ACE2 Polymorphisms
Median age (range)—yrs	62.1 (50.9–80.4)		
Male sex—no. (%)	4 (66.6)		
Moderate	1 (16.6)	13	0
Severe	3 (50.0)	18	4
Critical	2 (33.3)	4	1
Hospitalized no. (%)	6 (100)		

**Table 2:** ACE-related polymorphisms.

Locus	Type	Region	Reference Allele	Found Allele	COVID-19 Severity			Participants
					Moderate	Severe	Critical	
chr17:61556298	SNV	Intron	C	G/G	1	1	0	S03; S06
chr17:61557200	SNV	Exon	C	C/T	0	0	1	S05
chr17:61559923	SNV	Exon	C	C/T	1	1	0	S03; S06
chr17:61562309	SNV	Intron	C	C/T; T/T	1	1	0	S03; S06
chr17:61562490	INDEL	Intron	A	A/AG	0	1	0	S03
chr17:61562553	SNV	Intron	G	G/A; A/A	1	1	0	S03; S06
chr17:61562774	SNV	Intron	T	T/C; C/C	1	1	0	S03; S06
chr17:61564052	SNV	Exon	A	A/G; G/G	1	1	0	S03; S06
chr17:61564522	SNV	Intron	T	T/C; C/C	1	2	0	S02; S03; S06
chr17:61565990	SNV	Intron	G	G/C; C/C	1	1	0	S03; S06
chr17:61565998	SNV	Intron	A	A/C; C/C	1	1	0	S03; S06
chr17:61566031	SNV	Exon	G	G/A; A/A	1	1	0	S03; S06
chr17:61571516	INDEL	Intron	AGT	AGT/A	1	3	2	S01; S02; S03; S05; S06; S08
chr17:61573761	SNV	Exon	T	T/C; C/C	1	1	0	S03; S06
chr17:61574442	INDEL	Intron	ACCCTTGCCCTGCCCTGCCCA	ACCCTTGCCCTGCCCTGCCCA/ACCCTTGCCCTGCCCA	0	1	0	S02
chr17:61574446	INDEL	Intron	TTGCCC	TTGCCC/T	0	0	1	S05
chr17:61574492	SNV	Intron	G	G/A; A/A	1	1	0	S03; S06
				Total Events	13	18	4	

**Table 3:** ACE2-related polymorphisms.

Locus	Type	Region	Reference Allele	Found Allele	COVID-19 Severity			Participants
					Moderate	Severe	Critical	
chrX:15582265	SNV	Exon	G	A/A	0	1	0	S02
chrX:15589725	SNV	Intron	C	G/G	0	1	0	S02
chrX:15589925	INDEL	Intron	CAAAAAAAG	CAAAAAAAG/CAAAAAAA	0	1	1	S02; S05
chrX:15610348	SNV	Intron	C	T/T	0	1	0	S05
				Total Events	0	4	1	

## Data Availability

The datasets generated during and/or analyzed during the current study are not publicly available as this manuscript is part of the author F.A.G.’s PhD thesis but are available from the corresponding author on reasonable request.
